# The impact of retirement on loneliness in Europe

**DOI:** 10.1038/s41598-024-74692-y

**Published:** 2024-11-14

**Authors:** Sophie Guthmuller, Dörte Heger, Johannes Hollenbach, Anna Werbeck

**Affiliations:** 1 Health Economics and Policy Group, Department of Socioeconomics, Vienna University of Economics and Business, Welthandelsplatz 1, Building D4, 1020 Vienna, Austria; 2 RWI – Leibniz Institute for Economic Research, Essen, Germany; 3 Leibniz Science Campus Ruhr, Essen, Germany; 4 Paderborn University, Paderborn, Germany; 5 Ruhr University Bochum, Bochum, Germany

**Keywords:** Loneliness, Social isolation, Retirement, Instrumental variable, Causal effect, Risk factors, Human behaviour, Health policy

## Abstract

This article investigates the short- and long-term impacts of retirement on loneliness using panel data from the Survey of Health, Aging, and Retirement in Europe. To identify causal effects, we exploit differences in retirement rules across and within countries and use retirement thresholds in an instrumental variable setting. On average, we find that entering retirement leads to a reduction in loneliness in the long run and no clear effect in the short run. The reduction is driven by individuals being less likely to feel socially isolated and lacking companionship. Our results suggest that individuals adapt to retirement by increasing their activity levels and reap the benefits in terms of reduced loneliness and social isolation. The heterogeneity analysis shows that this is particularly true among the higher educated. The heterogeneity analysis also reveals that retirement increases feelings of loneliness for women in the short term and that the effect seems to be driven by women lacking companionship when their partner is not yet retired.

## Introduction

Loneliness and social isolation are important determinants of well-being. As such they have started receiving the full attention of policymakers^1,2^. Chronic loneliness is linked to a range of adverse outcomes: People experiencing repeated states of loneliness have an increased risk of ill-health, both physical and mental, and all-cause mortality^3–17^. Loneliness is also an economic issue. Lonely individuals have a higher demand for health care services^18^ and higher social service needs^19^.

In Europe, it is the elderly who are at the highest risk of loneliness^20^. This is particularly salient since Europe already has a large population of senior citizens and is growing older still. With increasing life expectancy and low birth rates, the proportion of elderly individuals is expected to increase further in the years ahead^21^. Policymakers have generally reacted to these demographic trends and the subsequent need to ensure funding for public pension schemes by postponing entry into retirement. Retirement, as a significant life event at older age, has wide-reaching consequences for individuals. The causal impact of retirement and the increases in pension eligibility ages on various (mental) health outcomes are well covered in the literature^22–30^. However, scarce evidence exists on the causal effect of retirement on loneliness^31,32^.

The direction of the impact of retirement on loneliness may be ambiguous. As retirement involves an abrupt end of social contact at work and fewer interactions with colleagues, retirement could lead to a reduction of social interactions followed by an increase in loneliness. While retirement does not seem to affect the social network size and its closeness as a whole^33,34^, previous studies have found that retirement causes a significant reduction in the share of colleagues within older adults’ social network^34–37^. Retirement may also lead to an increase in time spent with friends and family members, increasing the number or frequency of social contacts that may reduce loneliness. Similarly, as the share of family ties within a social network increases as a result of entering retirement^34^, the quality of social connections might increase, resulting in feeling less lonely. For example, Shin et al. 2020 find evidence that social support from the family significantly moderates the negative effect of involuntary retirement on loneliness in the US^38^.

The effect of retirement on loneliness may also be driven by how well individuals can find pastimes and activities to fill their lives, for instance through social activities. Previous studies have found that social participation is a large protective factor against loneliness among older adults, even for those with low socioeconomic status^39^. Likewise, Salm, et al. 2021^40^ find that taking care of grandchildren completely offsets the negative effect of retirement on mental health. The effect of retirement on loneliness may evolve over time. Retirees may feel (more) lonely during the transitional period of retirement, until they adapt to their new status, re-organize their life, and enter into a content retirement routine^41^. In this case, the effect of retirement causes situational loneliness in which retirees think their feelings of loneliness are temporary and will resolve^42,43^. Retirees may also experience a honeymoon period just after retirement but feel (more) lonely afterward because retirement does not meet their expectations, which could then lead to chronic loneliness with feelings of hopelessness^41,42^.

Education might affect how well individuals adapt or prepare for post-retirement life. Larger resources accumulated during the life course, and the capacity to anticipate life events are among the factors that could lead to different feelings of loneliness after retirement by educational levels^31,44,45^. Further, prior research has pointed to the gendered nature of work and retirement^26,46^ as well as mental health conditions^47^. Women have different preferences for social connections in retirement^46^, which may affect how lonely they end up feeling after retirement and how well they adjust to their new life situation.

Using data from the Survey of Health, Ageing and Retirement in Europe (SHARE), we contribute to the literature by exploiting differences in pension eligibility rules across and within European countries in an instrumental variable framework. This allows us to disentangle endogenous retirement behavior and identify the causal effect of retirement on loneliness. To analyze how the effect evolves over time, we examine both short-term (immediately after retirement) and long-term (four to six years after retirement) effects. While we find no clear effect of retirement on loneliness in the short term, our results show a significant reduction in loneliness because of retirement in the long term. To understand these results in more detail, we estimate the effect of retirement on feeling isolated, feeling left out, and lacking companionship. These components are captured by the dimensions of the loneliness scale and reflect different aspects of intimate loneliness^48^. Loneliness is an “unacceptable lack of (quality of) certain relationships”^42^. In this three-item scale, the quantity and the quality of social interactions seem to be captured most by isolation^49^, the quality or intimacy of social connections by lacking companionship^50^. We find a long-term reduction in isolation after retirement, indicating a rise in social interactions. In line with this result, a further analysis shows a long-term increase in the number of activities and group activities in retirement. This long-term reduction is present for both men and women. Generally, we see that women’s loneliness levels are particularly affected by the transition to retirement. This is evident in the companionship dimension capturing the intimacy (quality) of social connections. We do not find any significant effects for men. However, women are likely affected to a larger extent from a lack of companionship in the short-term and less in the long-term. A couple analysis shows that this short-term increase and long-term decrease in lack of companionship is especially present for women whose partner is still working when they retire.

The remainder of the paper is structured as follows. Section "Results" presents the results of the short- and long-term causal effects of retirement of loneliness and provides evidence of potential mechanisms. Section "Discussion" discusses our findings. Section "Methods" describes the data and outlines the empirical strategy.

## Results

### The impact of retirement on loneliness

Table 1 displays our results estimating the effect of retirement on loneliness and its dimensions. We show the endogenous FE-model from Eq. (1) as well as 2SLS results (FE-IV). We report both our short and long-term results and the joint first stage for the IV regression. The results for the endogenous FE estimates in column (1) suggest no effect of retirement on loneliness, neither in the short- nor in the long-term. Our short run 2SLS estimates in column (2) show a positive sign, suggesting that retirement might increase loneliness right after retiring. Still, this effect is not statistically significant. On the contrary, we find that retiring decreases loneliness in the long-term. The estimate is statistically as well as economically significant. The reduction corresponds to approximately a quarter of a standard deviation of the short three-item version of the Revised UCLA (R-UCLA) Loneliness Scale^51^.

**Table 1:** Estimates of the effect of retirement on (the dimensions of) loneliness.

	(1)	(2)	(3)	(4)	(5)	(6)	(7)	(8)
	Loneliness		Isolated		Left out		Lack companionship	
	FE	FE-IV	FE	FE-IV	FE	FE-IV	FE	FE-IV
Retired ${R}_{t}$	-0.016	0.135	0.003	0.039	-0.025^**^	0.012	-0.005	0.031
*(short-term)*	(0.030)	(0.126)	(0.010)	(0.042)	(0.011)	(0.044)	(0.013)	(0.054)
Retired ${R}_{t-1}$	-0.001	-0.300^***^	-0.007	-0.114^***^	0.014	-0.052	0.002	-0.086^*^
*(long-term)*	(0.032)	(0.095)	(0.011)	(0.030)	(0.012)	(0.039)	(0.014)	(0.055)
First stage	-	0.271^***^	-	0.271^***^	-	0.271^***^	-	0.271^***^
		(0.021)		(0.021)		(0.021)		(0.021)
First stage F	-	173.63	-	173.63	-	173.63	-	173.63
# Observations	39,398	39,398	39,398	39,398	39,398	39,398	39,398	39,398
# Individuals	19,699	19,699	19,699	19,699	19,699	19,699	19,699	19,699

Note: Estimates of the effect of retirement in the short- and longer-term (separate regressions). Column (1) shows estimates of the endogenous fixed effect regressions (FE), and column (2) shows the results of the fixed effect 2SLS regressions (FE-IV), our main results. Columns (3) and (4) show estimates of the effect of retirement on the probability of feeling isolated most or all of the time, and columns (5) and (6) on the probability of feeling left out most or all of the time, and columns (7) and (8) on the probability of feeling a lack of companionship most or all of the time. All regressions include control variables from Eqs. (1) and (2). Standard errors clustered at the individual level and at the policy level (age*gender*country) are in parentheses. *** *p* < 0.01, ** *p* < 0.05, * *p* < 0.1.0.

Our first-stage estimate, i.e., the estimate of the effect of the statutory retirement eligibility on self-reported retirement, is large and highly statistically significant, suggesting that the instrument is relevant. The F-statistic of the excluded instrument exceed the well-established thresholds of Stock and Yogo^52^ and Olea and Pflueger^53^ by a large margin. As a result, we argue that in line with existing studies, statutory pension eligibility rules are a strong instrument for retirement.

Next, we estimate the effect of retirement on the three dimensions of the loneliness scale separately. As they reflect qualitative, quantitative, and situational aspects of loneliness, we aim to understand different pathways through which retirement affects loneliness. Our FE model suggests very small and mostly statistically insignificant effects of retirement on the three dimensions, both in the short- and long-term. The IV estimates suggest small increases in all dimensions of loneliness in the short run after retirement. However, the estimates are not statistically significant. We find that in the long-term retiring causes the probability of feeling isolated and lacking companionship to decrease by 11.4 and 8.6 percentage points, respectively. The former estimate is statistically significant at the 1%, the latter at the 10% level. This indicates that both the quantity of social interactions (isolation) but also the quality of social connections (lack of companionship) of loneliness improves after retirement, but improvements take time.

Next, we study if and how education shapes the effect of retirement on loneliness levels. Table 2 shows the FE-IV estimates for the separate analysis by low and high education, where low (high) education is defined as below (above) median years of education. We find mostly positive but small and insignificant effects for low and high educated in the short run. In the long run, we find negative effects for loneliness and all subdimensions for both groups, which are significant in the high-educated group for overall loneliness and feeling isolated. As such, especially high-educated individuals seem to experience an improvement in feelings of loneliness and isolation after adaption to retirement.

**Table 2:** IV estimates of the effect of retirement on (dimensions of) loneliness by education.

	(1)	(2)	(3)	(4)	(5)	(6)	(7)	(8)
	Loneliness		Feeling isolated		Feeling left out		Lack companionship	
	Low educated	High educated	Low educated	High educated	Low educated	High educated	Low educated	High educated
Retired ${R}_{t}$	0.015	0.209	-0.017	0.076*	-0.064	0.058	0.044	0.021
(short-term)	(0.191)	(0.150)	(0.061)	(0.046)	(0.064)	(0.055)	(0.085)	(0.070)
Retired ${R}_{t-1}$	-0.190	-0.341***	-0.084	-0.125***	-0.009	-0.068	-0.116	-0.056
(long-term)	(0.161)	(0.124)	(0.056)	(0.035)	(0.059)	(0.048)	(0.082)	(0.069)
First stage	0.263***	0.275***	0.263***	0.275***	0.263***	0.275***	0.263***	0.275***
	(0.029)	(0.021)	(0.029)	(0.021)	(0.029)	(0.021)	(0.029)	(0.021)
First-stage F	82.19	173.68	82.19	173.68	82.19	173.68	82.19	173.68
# Observations	17,444	21,954	17,444	21,954	17,444	21,954	17,444	21,954
# Individuals	8,722	10,977	8,722	10,977	8,722	10,977	8,722	10,977

Note: Estimates of the effect of retirement in the short- and longer-term (separate regressions). Low (high) educated is defined as below (above) median years of education. All regressions include control variables from Eqs. (1) and (2). Standard errors clustered at the individual level and at the policy level (age*education*country) are in parentheses. *** *p* < 0.01, ** *p* < 0.05, * *p* < 0.1.

In a further analysis, we account for the gendered nature of retirement^46^ and mental health^47^. Table 3 shows the IV estimates of the separate analysis by gender. Our results in column (1) suggest that women feel lonelier shortly after entering retirement but are feeling less lonely in the long term. The increase and subsequent reduction are roughly equal in size and both effects are statistically significant at the 10% level. We do not find any statistically significant effects for men.

**Table 3:** IV estimates of the effect of retirement on (dimensions of) loneliness by gender.

	(1)	(2)	(3)	(4)	(5)	(6)	(7)	(8)
	Loneliness		Isolated		Left out		Lack companionship	
	Men	Women	Men	Women	Men	Women	Men	Women
Retired ${R}_{t}$	-0.099	0.336*	0.004	0.070	-0.048	0.064	-0.098	0.141*
(short-term)	(0.138)	(0.204)	(0.062)	(0.053)	(0.048)	(0.072)	(0.063)	(0.084)
Retired ${R}_{t-1}$	-0.226	-0.356***	-0.126**	-0.101***	-0.040	-0.064	-0.007	-0.156**
(long-term)	(0.141)	(0.128)	(0.046)	(0.041)	(0.062)	(0.053)	(0.065)	(0.083)
First stage	0.266***	0.274***	0.266***	0.274***	0.266***	0.274***	0.266***	0.274***
	(0.024)	(0.031)	(0.024)	(0.031)	(0.024)	(0.031)	(0.024)	(0.031)
First-stage F	119.89	75.38	119.89	75.38	119.89	75.38	119.89	75.38
# Observations	16,688	22,710	16,688	22,710	16,688	22,710	16,688	22,710
# Individuals	8,344	11,355	8,344	11,355	8,344	11,355	8,344	11,355

Note: Estimates of the effect of retirement in the short- and longer-term. All regressions include control variables from Eqs. (1) and (2). Standard errors clustered at the individual level and the policy level (age*gender*country) are in parentheses. *** *p* < 0.01, ** *p* < 0.05, * *p* < 0.1.

Women and men both feel significantly less isolated in the long term. While no effect of retirement is found on the indicator for feeling “left out” for either men or women, retirement increases the feeling of lacking companionship for women in the short term and decreases this feeling in the long term. We find no such effect for men.

### Potential mechanisms

Next, we turn to potential mechanisms that might explain the impact of retirement on loneliness and its dimensions. One potential mechanism may be the participation in (group) activities which can increase the quantity and frequency of social interactions, and thus the size of the social network^15^. To test this mechanism, we estimate the effect of retirement on the number of activities undertaken in the previous year and the probability of having participated in a group activity in the previous year (Tables 4 and 5). We find statistically significant positive long-term estimates that are driven by the group of high-educated (Table 4) but are similar for both men and women (Table 5). In the long run, compliers start 0.5 new activities per year, on average, after retiring. Furthermore, retirement seems to have a positive effect on the likelihood of participating in a group activity. The heterogeneity analysis shows again that the effect is explained by the group of high-educated (Table 4) and the effect does not differ by gender (Table 5). This indicates a rise in social interactions. It may even point towards a potential mechanism for how the adjustment to life in retirement works.

**Table 4:** Retirement and channels by Education.

	(1)	(2)	(3)	(4)	(5)	(6)
	Number of activities			Participate in group activity		
	All	Low educated	High educated	All	Low educated	High educated
Retired ${R}_{t}$	-0.0759	-0.035	-0.111	0.0230	0.023	0.018
(short-term)	(0.102)	(0.192)	(0.141)	(0.0456)	(0.085)	(0.052)
Retired ${R}_{t-1}$	0.516***	0.280	0.678***	0.128***	0.023	0.199***
(long-term)	(0.121)	(0.225)	(0.165)	(0.0429)	(0.093)	(0.059)
First stage	0.271***	0.263***	0.275***	0.271***	0.263***	0.275***
	(0.021)	(0.029)	(0.021)	(0.021)	(0.029)	(0.021)
First-stage F	173.63	82.39	177.10	173.63	82.39	177.10
# Observations	39,398	17,444	21,954	39,398	17,444	21,954
# Individuals	19,699	8,722	10,977	19,699	8,722	10,977

Note: Estimates of the effect of retirement in the short- and long-term. Low (high) educated is defined as below (above) median years of education. All regressions include control variables based on Eqs. (1) and (2). Standard errors clustered at the individual level and at the policy level (age*gender*country) are in parentheses. *** *p* < 0.01, ** *p* < 0.05, * *p* < 0.1.

**Table 5:** Retirement and channels by gender.

	(1)	(2)	(3)	(4)	(5)	(6)
	Number of activities			Participate in group activity		
	All	Men	Women	All	Men	Women
Retired ${R}_{t}$	-0.0759	-0.180	0.005	0.0230	0.035	0.016
(short-term)	(0.102)	(0.162)	(0.128)	(0.0456)	(0.067)	(0.062)
Retired ${R}_{t-1}$	0.516***	0.543***	0.511***	0.128***	0.128**	0.129**
(long-term)	(0.121)	(0.181)	(0.159)	(0.0429)	(0.064)	(0.059)
First stage	0.271^***^	0.266^***^	0.274^***^	0.271^***^	0.266^***^	0.274^***^
	(0.021)	(0.024)	(0.031)	(0.021)	(0.024)	(0.031)
First-stage F	173.63	119.89	75.38	173.63	119.89	75.38
# Observations	39,398	16,688	22,710	39,398	16,688	22,710
# Individuals	19,699	8,344	11,355	19,699	8,344	11,355

Note: Estimates of the effect of retirement in the short- and long-term. All regressions include control variables based on Eqs. (1) and (2). Standard errors clustered at the individual level and at the policy level (age*gender*country) are in parentheses. *** *p* < 0.01, ** *p* < 0.05, * *p* < 0.1.

Another potential mechanism might be more time spent with family members resulting in strengthened family ties and as such an increase in intimate social connections. This mechanism might be particularly important since the decision to retire is usually not only an individual but a household decision^54–57^. In an additional analysis, we therefore focus on couples. We estimate the effect of retirement on loneliness and its dimensions for individuals retiring between waves 5 and 6 whose partner is *not* retired in wave 6. The results are shown in Table 6. Men have a higher number of observations in this couple analysis (even though there are more women in our overall sample) since they tend to retire earlier than their, on average, younger partners and consequently have a partner who is not retired at wave 6. In Table 7, we analyze the effect of retirement on loneliness for individuals whose partner is retired in wave 6.

**Table 6:** IV estimates of the effect of retirement on (dimensions of) loneliness if the partner is not retired in wave 6, by gender.

	(1)	(2)	(3)	(4)	(5)	(6)	(7)	(8)
	Loneliness		Isolated		Left out		Lack of companionship	
	Men	Women	Men	Women	Men	Women	Men	Women
Retired ${R}_{t}$	0.144	0.808*	0.106	0.138	0.005	0.389*	-0.122	0.325*
*(short-term)*	(0.260)	(0.477)	(0.089)	(0.196)	(0.086)	(0.227)	(0.110)	(0.190)
Retired ${R}_{t-1}$	-0.540	-0.916**	-0.239**	-0.242**	-0.099	-0.312*	-0.125	-0.359*
*(long-term)*	(0.336)	(0.393)	(0.099)	(0.105)	(0.100)	(0.181)	(0.134)	(0.202)
First stage	0.275***	0.302***	0.275***	0.302***	0.275***	0.302***	0.275***	0.302***
	(0.032)	(0.061)	(0.032)	(0.061)	(0.032)	(0.061)	(0.032)	(0.051)
First-stage F	75.73	24.59	75.73	24.59	75.73	24.59	75.73	24.59
# Observations	5,308	3,602	5,308	3,602	5,308	3,602	5,308	3,602
# Individuals	2,654	1,801	2,654	1,801	2,654	1,801	2,654	1,801

Note: Estimates of the effect of retirement in the short- and long-term. All regressions include control variables form Eqs. (1) and (2). Standard errors clustered at the individual level and at the policy level (age*gender*country) are in parentheses. *** *p* < 0.01, ** *p* < 0.05, * *p* < 0.1.

**Table 7:** IV estimates of the effect of retirement on (dimensions of) loneliness if the partner is retired in wave 6, by gender.

	(1)	(2)	(3)	(4)	(5)	(6)	(7)	(8)
	Loneliness		Isolated		Left out		Lack companionship	
	Men	Women	Men	Women	Men	Women	Men	Women
Retired ${r}_{t}$	0.002	0.192	-0.070	-0.026	-0.038	0.069	0.028	0.160
(short-term)	(0.288)	(0.273)	(0.089)	(0.101)	(0.112)	(0.112)	(0.157)	(0.128)
Retired ${r}_{t-1}$	-0.169	-0.105	0.008	0.000	-0.061	-0.026	-0.107	-0.136
(long-term)	(0.178)	(0.274)	(0.072)	(0.105)	(0.101)	(0.124)	(0.110)	(0.163)
# Observations	5,280	7,370	5,280	7,370	5,280	7,370	5,280	7,370
# Individuals	2,640	3,685	2,640	3,685	2,640	3,685	2,640	3,685
First stage	0.241***	0.217***	0.241***	0.217***	0.241***	0.217***	0.241***	0.217***
	(0.032)	(0.025)	(0.032)	(0.025)	(0.032)	(0.025)	(0.032)	(0.025)
First-stage F	57.16	75.20	57.16	75.20	57.16	75.20	57.16	75.20

Note: Estimates of the effect of retirement in the short- and long-term on the loneliness scale, and its dimensions, by gender separately and restricted to individuals whose partner is retired in wave 6. All regressions include control variables based on Eqs. (1) and (2). Standard errors clustered at the individual level and at the policy level (age*gender*country) are in parentheses. *** *p* < 0.01, ** *p* < 0.05, * *p* < 0.1.

Again, the results in Table 6 show that retirement affects particularly women’s loneliness levels. Retirement does not seem to affect men – whose partner is not retired with them in wave 6 – in the short-term and only slightly in the long-term, which is driven solely by their decrease in isolation. Women in the same situation are instead affected across time and dimensions. In the short-term, retirement increases their loneliness level in all dimensions but isolation. In the long term, retirement decreases it in all dimensions without exception. Interestingly we do not find any significant effects when we consider individuals in a partnership whose partners have retired before or along with them in wave 6 (Table 7). Potentially, the decision to jointly retire alleviates any adverse effects on loneliness since retirement is experienced together with one’s partner.

With this in mind, the results for lack of companionship are particularly interesting. Companionship captures the quality of (close) social connections and the enjoyment of spending time with someone close, such as one’s partner. We see a short-term increase in lacking companionship for women when the partner is not retired, and thus spends less time with him or her, and a long-term decrease when the partner might have retired between waves 6 and 8 (about two-thirds of the women’s partner that had not retired in wave 6, had retired in wave 8 in our sample).

## Discussion

We examine the short- and long-term effects of retirement on loneliness using data from 13 European countries and Israel. By exploiting differences in retirement eligibility rules between and within countries, we account for the endogenous nature of the retirement decision. Our results suggest that while there is no clear average short-term effect of retirement on loneliness, retirement leads to a significant reduction in loneliness in the long-term. The reduction corresponds to approximately a quarter of the standard deviation of the short three-item version of the Revised UCLA Loneliness Scale^51^. These results are driven by individuals being less likely to feel socially isolated or less likely to feel a lack of companionship some years after retiring. This hints at improvements both in the quantity and quality of social interactions.

The heterogeneity analysis shows that these results are driven by the high-educated group and that retirement affects women’s loneliness levels much more than men’s and in more dimensions. Women experience an increase in overall loneliness shortly after retirement and a decrease after a few years of retirement. This is due to an increase in lack of companionship (decrease in quality of social connections) shortly after entering retirement, which is reversed in the long-term. Interestingly, our couple analysis shows that this is apparent only for women whose partner is still in the labor force when they enter retirement. Furthermore, the high-educated, and both women and men experience a long-term decrease in feeling socially isolated. Indeed, we find that retiring causes the high-educated and, both men and women to participate in more activities and be more likely to participate in group activities in the long-term.

Our results suggest that individuals are likely to adapt to life in retirement and, as a result, feel more socially connected and less lonely after several years. As such, we contribute to the literature on the mental health effects of retirement. Previous evidence on the effect of retirement on mental health is mixed. Some studies find no effect^23,58,59^, while others find a positive impact (for certain groups)^25,27,60,61^. We run additional regressions with depression (EURO-D scale) as the dependent variable using our sample. In line with our main findings, the results show a null effect of retiring on depressive symptoms in the short run and a decrease in depressive symptoms in the long run. Interestingly, this effect is driven by men. There are no differential effects by education. The results are available from the authors upon request.

At a time when policymakers are increasingly focusing on loneliness as a distinct health issue with far-reaching implications, our findings contribute to a better understanding of how retirement policies affect the well-being of seniors. While concerns about the financial stability of the social security systems are usually at the center of the debate and a strong argument towards delaying retirement, we also highlight the benefits of retirement, at least when people remain socially active. Retirees can potentially benefit greatly from policies aimed at maintaining or even increasing their social inclusion. In terms of public policy, this can include ensuring better opportunities for retirees to work part-time or volunteer and ensuring age-friendly public infrastructure. Local authorities can contribute, for example, by maintaining inclusive public spaces.

## Methods

### Data

Our paper uses data from SHARE, a longitudinal survey of individuals aged 50 or older conducted in Europe and Israel^62^. This data set provides individual-level microdata on health, socio-economic status, and social and family networks that are comparable across countries. We use data from waves 5 (collected in 2013), 6 (collected in 2015), and 8 (collected in late 2019 and early 2020)^63–65^ in which the UCLA revised loneliness scale (our dependent variable) was collected. Overall, 14 countries participated in each of the three waves (Austria, Belgium, Czech Republic, Denmark, Estonia, France, Germany, Italy, Luxembourg, Slovenia, Spain, Sweden, Switzerland, and Israel). Further, we obtain information on retirement rules from the European Commission’s Mutual Information System on Social Protection^66^ as well as Israel’s National Insurance Institute^67^.

#### Variables

Loneliness, our main dependent variable, is defined as “a situation experienced by the individual as one where there is an unpleasant or inadmissible lack of (quality of) certain relationships. This includes situations in which the number of existing relationships is smaller than is considered desirable or admissible, as well as situations where the intimacy one wishes for has not been realized”^42^. Loneliness could then include (subjective) social isolation — the (perceived) “lack of, or deficit in, the quantity of a social network”^43^, and emotional isolation — “the lack of person(s) to whom one feels attached”^43^.

In our data, loneliness is measured by the short three-item version of the Revised UCLA Loneliness Scale^51,68–70^. This measure of loneliness takes into account the multidimensionality of the feelings of loneliness with a scale based on responses to three questions: “How much of the time do you feel …” (1) “…you lack companionship?”, (2) “…left out?”, and (3) “…isolated from others?”. For each of these questions, participants choose between “often”, “some of the time” and “hardly ever or never”, which yield three, two, and one points, respectively. The scores are added up to calculate the loneliness scale, which thus ranges from 3 (not lonely) to 9 (very lonely), and which serves as our measure of overall loneliness. The short version of the Revised UCLA Loneliness Scale has been shown to perform as well as the 20-item-scale to measure the distribution in the feelings of loneliness in the older population^69,70^.

Besides the overall level of loneliness, we are interested in the different dimensions of loneliness, i.e., the three components of the loneliness scale, as a first step to exploring potential sources of loneliness. Feeling isolated addresses the discrepancy between one’s desired and one’s actual quantity and quality of social interactions^49^. Lack of companionship is concerned with the discrepancy between one’s desired and one’s actual quality social (or intimacy) of social connections^50^. Feeling left out is usually the result of feeling excluded from a group of friends or family members in certain situations and relates to the discrepancy between one’s desired and one’s actual quality or quantity of collective connections^48,51,70^. We construct categorical variables for each of the dimensions of the loneliness scale which are equal to one if the respondent reported feeling a lack of companionship/left out/isolated from others sometimes or often, and zero otherwise.

As a further step to explore sources of loneliness, we use two different outcomes to measure leisure and activities. The first is the number of different types of activities that respondents participated in the year prior to the interview. This measure combines several specific types of activities, such as volunteering or charity work, attending educational training or courses, sports, political or community events, reading, or playing different types of games (word or number games/cards or board games). In addition, we construct a dummy variable equal to one if an individual has participated in a group activity and zero otherwise. We define group activities as volunteering or charity work, attending an education or training course, attending a sports, social, or other type of club, taking part in the activities of a religious, political, or community organization, and playing cards or board games.

We construct our retirement variable based on three definitions. According to our main definition, individuals are classified as retired (the ‘treatment’ group) if they report being retired at the time of the interview. This definition is similar to Heller-Sahlgren^27^. The control group thus consists of individuals who are working, unemployed, homemakers, permanently ill or disabled, or engaged in other activities. Although not all individuals in the control group are in the labor force, reaching the statutory retirement age may affect them in a similar way to those in the work force since retirement may remove the pressure to find a job or to justify not working to oneself or others^71^. Our second definition of retirement additionally includes homemakers, those who are permanently ill or disabled, and those engaged in other activities if they have not done any paid work in the last four weeks, as these individuals are unlikely to re-enter the labor force shortly before retirement^72^. The control group for this definition is therefore unemployed, employed, or self-employed individuals. This second definition is close to the one used by Coe and Zamarro^23^, except that Coe and Zamarro count unemployed individuals as retired. In the third definition, analogous to the first definition, we define as retired individuals who report being retired at the time of the interview. The control group, however, is restricted to employed individuals. We consider respondents to be in the labor force if they report being employed, self-employed, or not retired and have done any paid work in the past four weeks, while excluding homemakers, the permanently ill or disabled, and those doing other work. In this way, we can only compare retired people with those who are still working. The first definition is used as baseline specification, and the two alternative definitions serve as robustness checks.

Further, gender and education, where low (high) education is defined as below (above) the median years of education, are used in the heterogeneity analysis. We also use information on age, gender, the number of children, and country of residence in each wave to define retirement eligibility.

#### Sample

Our sample uses observations from all 14 countries and waves 5, 6, and 8 of SHARE. We include individuals between the ages of 50 and 80 at the time of the wave 6 interview (2015). This covers around 10 years below and above the youngest and oldest age retirement rules (see Table A1 in the appendix). We exclude individuals with missing information in the variables described in the variables section and individuals who report “undoing” their retirement, i.e., report being retired at one wave and claim not to be retired at a later wave. This dataset includes 58,547 individuals. Our study design allows us to estimate long-term effects, however, it requires balanced panel data. Therefore, we only use individuals who are present in all three waves. The final dataset is a balanced panel of 19,699 individuals (59,097 observations). Most of the observations lost when balancing the dataset are individuals who participated in waves 5 and 6 but did not participate in wave 8 (30% of the sample). The main reason for this is that SHARE stopped collecting data for wave 8 in March 2020 due to Covid-19. As a result, fewer people were surveyed in wave 8 than in waves 5 and 6. To address a potential attrition bias, we run robustness checks using inverse probability weighting. The results are reported in Table A8 in the appendix and are discussed in the robustness checks section.

We use the eligibility for statutory retirement as an instrument for retiring. Standard retirement ages vary by country, gender, birth cohort (France, Germany, Israel), and the number of children (Czech women). The earliest and latest statutory retirement ages in our sample are 57.3 and 67, respectively. An overview of all official retirement ages by country and gender for the years of interest is provided in Table A1 in the appendix.

Summary statistics by wave and retirement status are shown in Table 8 and Table A2 in the appendix. Average loneliness levels increase by wave, both in the entire sample as well as among retired and non-retired individuals. There are no significant differences between individuals who are retired and those who are not retired. Mean loneliness levels are higher among women compared to men, and among low-educated compared to high-educated. Average loneliness increases over the waves, i.e., over time. Changes over time are precisely what our study design is picking up.

**Table 8:** Summary statistics.

	Whole sample		Men		Women		Low educated		High educated	
	Not Retired	Retired	Not Retired	Retired	Not Retired	Retired	Not Retired	Retired	Not Retired	Retired
*Wave 5*										
Age	58.78	68.80	58.57	69.08	58.91	68.58	60.01	69.29	57.99	68.33
Female	0.60	0.55	0.00	0.00	1.00	1.00	0.64	0.56	0.58	0.54
High educated	0.61	0.51	0.65	0.52	0.59	0.50	0.00	0.00	1.00	1.00
Loneliness	3.64	3.69	3.55	3.56	3.70	3.79	3.75	3.70	3.57	3.67
Feeling isolated	0.12	0.12	0.10	0.10	0.13	0.14	0.14	0.13	0.10	0.12
Feeling left out	0.16	0.18	0.15	0.14	0.17	0.20	0.18	0.17	0.15	0.18
Lack companionship	0.25	0.28	0.21	0.24	0.28	0.32	0.29	0.29	0.23	0.27
Number of activities	2.54	2.52	2.49	2.42	2.57	2.60	2.00	2.21	2.89	2.81
Participate in group activity	0.68	0.65	0.70	0.68	0.66	0.64	0.56	0.59	0.75	0.71
Observations	9,410	10,289	3,733	4,611	5,677	5,678	3,660	5,062	5,750	5,227
*Wave 6*										
Age	59.96	70.05	59.54	70.27	60.21	69.87	61.30	70.61	59.13	69.52
Female	0.62	0.55	0.00	0.00	1.00	1.00	0.65	0.56	0.59	0.54
High educated	0.62	0.52	0.66	0.53	0.59	0.50	0.00	0.00	1.00	1.00
Loneliness	3.71	3.74	3.61	3.61	3.78	3.85	3.82	3.77	3.64	3.72
Feeling isolated	0.13	0.14	0.12	0.11	0.14	0.16	0.16	0.14	0.12	0.13
Feeling left out	0.18	0.19	0.16	0.16	0.20	0.22	0.20	0.18	0.17	0.20
Lack companionship	0.28	0.30	0.24	0.25	0.31	0.34	0.32	0.32	0.26	0.29
Number of activities	2.54	2.53	2.52	2.42	2.55	2.61	1.99	2.21	2.88	2.82
Participate in group activity	0.67	0.65	0.69	0.66	0.65	0.64	0.53	0.58	0.75	0.72
Observations	7,876	11,823	3,027	5,317	4,849	6,506	3,013	5,709	4,863	6,114
*Wave 8*										
Age	63.38	72.87	62.39	73.09	63.92	72.69	65.34	73.67	62.17	72.19
Female	0.65	0.55	0.00	0.00	1.00	1.00	0.71	0.57	0.61	0.54
High educated	0.62	0.54	0.68	0.55	0.58	0.53	0.00	0.00	1.00	1.00
Loneliness	3.74	3.79	3.60	3.68	3.81	3.89	3.88	3.85	3.65	3.74
Feeling isolated	0.14	0.15	0.11	0.13	0.16	0.17	0.18	0.16	0.12	0.14
Feeling left out	0.19	0.20	0.16	0.18	0.20	0.22	0.20	0.21	0.18	0.20
Lack companionship	0.28	0.31	0.24	0.27	0.30	0.35	0.32	0.33	0.25	0.29
Number of activities	2.52	2.49	2.51	2.36	2.52	2.59	1.89	2.16	2.90	2.77
Participate in group activity	0.67	0.64	0.70	0.64	0.65	0.64	0.52	0.57	0.76	0.70
Observations	4,955	14,744	1,749	6,595	3,206	8,149	1,898	6,824	3,057	7,920

Notes: Means. The retired group includes everyone who is retired – by wave—according to retirement definition (1): all individuals who claim to be retired or who self-reported a retirement date predating the interview. The not-retired group includes everyone who is not retired according to this definition. Loneliness refers to the short version of the R-UCLA loneliness scale. Feeling isolated, feeling left out and lack companionship are indicator variables equal to one if respondents felt either of these feelings “some of the time” or “often”. The number of activities refers to a specific set of activities respondents have engaged in during the last 12 months. “Participate in group activities” refers to a dummy equal to one if the respondent has engaged in at least one kind of activity classified as a group activity during the last 12 months. Low (high) educated is defined as below (above) median years of education.

**Fig. 1:**
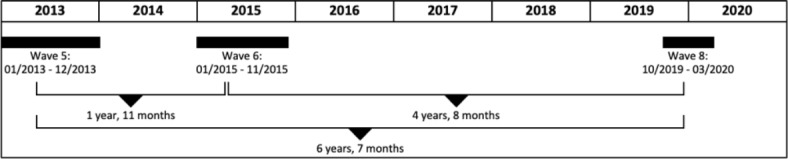
Data collection of SHARE and average time passed between interviews in our sample. Average time passed between interviews in waves, 5, 6, and 8 of SHARE. Source: Authors’ own representation based on SHARE.

Figure 1 shows interview intervals for waves 5, 6, and 8 of SHARE for individuals in our sample, as well as the time between each of the waves’ mean interview dates. We analyze individuals retiring between waves 5 and 6, an interval of about two years. To analyze short-term effects, we observe their change in loneliness levels between waves 5 and 6. For long-term effects, we observe their change in loneliness levels between waves 6 and 8 that occurs, on average, between about 4.5 years and 6.5 years after retiring. To ensure our long-term estimates are not driven by people retiring between waves 6 and 8, who are in our control group, we run a robustness check where these individuals are removed from the sample entirely.

### Empirical strategy

The long-term effect of retirement on loneliness can be estimated by a fixed effect (FE) estimation as:

1 $${L}_{i,t}={\gamma}_{0}+{\gamma}_{1}{R}_{i,t-1}+{\gamma}_{2}{AGE}_{i,t-1}+{\gamma}_{3}{AGE}_{i,t-1}^{2}+{\delta}_{m}+{\alpha}_{i}+{\epsilon}_{i,t}$$

where ${L}_{i,t}$ denotes the individual *i*’s level of loneliness (or our alternative outcome measures) at time *t*, ${R}_{i,t-1}$ is retirement status in wave $t-1$. Our estimation technique does not require additional control variables since it relies on the random variation in retirement behavior induced by pension policies. We include linear and quadratic age as well as a set of indicator variables for interview month (${\delta}_{m}$) to account for seasonal trends. Time-invariant individual effects are captured by ${\alpha}_{i}$. Unobserved influences on $L$ are captured by the error term $\epsilon$. We cluster standard errors at the individual level and at the retirement policy level (age in wave 5*gender*country)^73^.

The endogenous nature of the retirement decision may lead to several endogeneity problems. First, there may be reverse causality. An individual’s level of loneliness might influence their decision to retire. Second, we are not able to observe and control for all factors that are jointly relevant to loneliness and the retirement decision, such as other major life events like the death of a spouse or friend, adverse health shocks, or unobserved characteristics^25^. Therefore, to estimate causal effects, we use a source of exogenous variation in retirement behavior: Institutional retirement rules. Table A1 in the appendix reports the differences in age eligibility rules in the countries and waves included in the sample, for men and women separately. This instrument is highly relevant to predicting retirement but is unlikely to influence loneliness directly. With the same motivation, the instrument is widely applied to study the effect of retirement on life satisfaction^74,75^, general health^23,58,59^, mental health^27,40,59,61,76,77^, cognitive functioning^22,60,78–80^, health behavior^26,81^, and healthcare use^74^.

The decision to retire can be thought of as a dynamic incentive system in which the benefits of retirement are weighed against the benefits of remaining in the labor force^79^. Although some people choose to retire early, likely with reduced benefits, statutory retirement ages act as thresholds at which the probability of retiring increases dramatically as full pension eligibility is reached. Moreover, while individuals opting for early retirement benefits might be a select group of individuals who already have specific plans on how to spend their time once they are retired, individuals retiring upon reaching the statutory retirement age arguably better represent the average individual who has to adjust to being retired, which are the focus of this study. Therefore, we use the statutory retirement age as a source of exogenous variation in retirement behavior. Our instrument for retirement status is defined as being at or above the statutory retirement age of an individual`s country of residence.

To estimate the causal effect of retirement on loneliness we use a two-stage least squares (2SLS) estimation. The first stage estimates an exogenous variation in retirement behavior:

2 $${R}_{i,t-1}={\beta}_{0}+{\beta}_{1}ELI{G}_{i,t-1}+{\beta}_{2}{AGE}_{i,t-1}+{\beta}_{3}{AGE}_{i,t-1}^{2}+{\delta}_{m}+{\alpha}_{i}+{\zeta}_{i,t}$$

where $ELI{G}_{i,t-1}$, the instrument, is a dummy variable equal to one if an individual $i$ is eligible for retirement in $t-1$. The predicted values ${\widehat{R}}_{i,t-1}$ are then plugged into the second stage given by Eq. (1). This set-up allows us to estimate the long-term effect of retirement on loneliness, i.e., the effect of retiring, arising from the changes in retirement eligibility between wave 5 and wave 6 on the difference in loneliness level between wave 6 and wave 8. We use an instrumental variable approach and not a regression discontinuity, as we are interested in estimating both the short-term and long-term causal effects of retirement on loneliness^25,82^. 

Short-term effects of retirement on loneliness are estimated using this 2SLS setup with variables measured at $t$, instead of $t-1$, i.e. the effect of retiring, driven by the changes in pension eligibility, between wave 5 and wave 6 on the difference in loneliness level between wave 5 and wave 6.

The 2SLS estimation identifies a causal effect of retirement on loneliness if four standard IV assumptions hold: first stage, independence, exclusion restriction, and monotonicity. The first stage assumption refers to the relevance of the instrument, i.e., the instrument must be correlated with the treatment. Like many other studies using this set-up, we show that the first stage assumption is fulfilled as crossing the retirement eligibility threshold elicits a strong response in the probability of retirement (see our first stage results in Table 3). We include the Kleibergen-Paap rank Wald F statistic of the excluded instrument in the first stage (“first-stage F”) in our regression results^83^. Independence refers to the instrument being as good as randomly assigned, i.e., uncorrelated with the errors. This implies that becoming eligible for retirement must be exogenous to the level of loneliness. The exclusion restriction states that the instrument – retirement eligibility – must not affect loneliness directly, but only via its effect through the treatment, retirement. We argue that even though loneliness might be directly affected by many factors, simply becoming eligible for retirement is not one of them. Monotonicity requires that – while the instrument may not affect some – those who are affected, are affected in the same way. This assumption would be violated if some individuals chose to retire due to crossing the retirement eligibility threshold, while at the same time, other individuals chose to come out of retirement as a direct result of becoming eligible. Such defying behavior seems implausible. If these assumptions hold, our IV estimate identifies a local average treatment effect (LATE), i.e., the average effect of retirement on loneliness for the compliers who retire because they reach the statutory retirement age.

In addition to running the estimation on the full sample, we split our sample by gender and level of education. Further, we check the robustness of our results with respect to the definition of retirement, the specification of age (age fixed effects, cubic specification, country specific age trends), the age window of individuals in the dataset, and the composition of the control group.

### Robustness checks

We check the robustness of our results with respect to the definition of retirement, loneliness as a binary outcome, the age specification, the age window of individuals in the dataset, and the composition of the control group. Tables A3–A7 in the online appendix show the results of our robustness checks.

Using the additional retirement definitions as in Heller-Sahlgren^27^, i.e. when we include homemakers, those who are permanently ill or disabled, and those who reported being engaged in “other activities” as retired (definition 2, column 1 in Tables A3–A7) and comparing only those who are employed with those who report being retired (definition 3, column 2), the estimates are similar to the ones with our main specification.

Using a dummy variable as an outcome that equals one if an individual is above the lowest level of loneliness (> 3) in column (3) of Tables A3–A7, we find no effect in the short-term. But in the long run, retiring reduces the probability of being lonely by 11.2% points in the full sample. The gender analysis reveals similar results for women: Table A6, column 3 shows the results. We find a positive and statistically significant effect on the probability of feeling lonely in the short-term (14.2% points) and a significant negative long-term effect (17.7% points). Among men, our analysis shows a negative and statistically significant effect at the 10% level on the probability of feeling lonely short-term, and a negative but not statistically significant long-term effect (Table A7, column 3). The heterogeneity by education shows that the negative effect in the long-term is driven by the high-educated with a decrease of 13.9% points (column 3, Table A5).

The estimates using age dummies, an additional cubic polynomial of age, and country specific age trends, to more flexibly control for age are robust to the main specification (columns 4–6, respectively, in Tables A3–A7).

Following Heller-Sahlgren^27^ and using the earliest and latest statutory retirement ages of the analyzed countries as bounds, we construct different samples with narrower age ranges at wave 6: ages 52–72 (five years to/from the earliest/latest retirement age), 54–70 (+/- three years), and 56–68 (+/- one year). Using narrower age ranges does not qualitatively change the results (Tables A3–A7, columns 7–9).

We measure the impact of retirement on loneliness for individuals who have retired between waves 5 and 6 (the treatment group). The control group is composed of individuals that have never retired across the three waves, individuals that are always retired across the three waves, and individuals who retire between waves 6 and 8 (the later-retired). To check the robustness of our results to the composition of the control group, we run the estimation excluding each group of control individuals one at a time. We find that the exclusion of the never-retired (column 10 of Tables A3–A7), of the always-retired (column 11), or the exclusion of the later-retired (column 12), one at a time, do not qualitatively change our main results.

Additionally, to account for possible attrition, we include estimates of our main regressions with inverse probability weights for the whole sample as well as men and women separately in Table A8 in the appendix. To calculate the weights, we predict individual probabilities of not being included in wave 8 after being included in both waves 5 and 6 (our relevant potential source of attrition). Controls include retirement status, age, squared age, gender, physical health, marital status, level of education, and level of loneliness with time-varying variables measured in wave 5, i.e., before treatment. The inverse probability of predicted attrition according to this model is then used to weight our regressions. This may account for attrition under the assumption that attrition is random between treatment and control group given the observed characteristics. When using these weights, our main results remain unchanged and retain their qualitative interpretation (see Table A8).

## Electronic supplementary material

Below is the link to the electronic supplementary material.

Supplementary Material 1

## Data Availability

This paper uses data from SHARE Waves 5, 6, and 8 (DOIs: 10.6103/SHARE.w5.800, 10.6103/SHARE.w6.800, 10.6103/SHARE.w8.800), see Börsch-Supan, Brandt et al. (2013) for methodological details. The SHARE data collection has been funded by the European Commission through FP5 (QLK6-CT-2001-00360), FP6 (SHARE-I3: RII-CT-2006-062193, COMPARE: CIT5-CT-2005-028857, SHARELIFE: CIT4-CT-2006-028812), FP7 (SHARE-PREP: GA N°211909, SHARE-LEAP: GA N°227822, SHARE M4: GA N°261982) and Horizon 2020 (SHARE-DEV3: GA N°676536, SERISS: GA N°654221) and by DG Employment, Social Affairs & Inclusion. Additional funding from the German Ministry of Education and Research, the Max Planck Society for the Advancement of Science, the U.S. National Institute on Aging (U01_AG09740-13S2, P01_AG005842, P01_AG08291, P30_AG12815, R21_AG025169, Y1-AG-4553-01, IAG_BSR06-11, OGHA_04-064, HHSN271201300071C) and from various national funding sources is gratefully acknowledged. Data are publicly available for researchers (see https://www.share-project.org/)^62^.
