# Supplementary material for: The impact of retirement on loneliness in Europe
Source: Sci Rep. 2024 Nov 14;14:26971. doi: 10.1038/s41598-024-74692-y (PMC11555320; doi:10.1038/s41598-024-74692-y)
Supplement: Supplementary file 1 — Supplementary Material 1 [file 41598_2024_74692_MOESM1_ESM.docx]

# Online Appendix

Table A1: Official retirement ages by country, gender, and years of interest

|  | 2013 | | 2015 | | 2019 | | 2020 | |
| --- | --- | --- | --- | --- | --- | --- | --- | --- |
| Country | Men | Women | Men | Women | Men | Women | Men | Women |
| Austria | 65 | 60 | 65 | 60 | 65 | 60 | 65 | 60 |
| Belgium | 65 | 65 | 65 | 65 | 65 | 65 | 65 | 65 |
| Czech Republic | 62.5 | 57.33-61.33 | 62.83 | 58-62 | 63.5 | 59.17-63.17 | 63.67 | 59.67-63.67 |
| Denmark | 65 | 65 | 65 | 65 | 65.5 | 65.5 | 66 | 66 |
| Estonia | 63 | 62 | 63 | 62.5 | 63.75 | 63.75 | 63.75 | 63.75 |
| France | 60-62 | 60-62 | 60-62 | 60-62 | 60-62 | 60-62 | 62 | 62 |
| Germany | 65-67 | 65-67 | 65-67 | 65-67 | 65-67 | 65-67 | 65-67 | 65-67 |
| Israel | 67 | 62 | 67 | 62 | 67 | 62 | 67 | 62 |
| Italy | 66.25 | 62.25 | 66.25 | 63.75 | 67 | 67 | 67 | 67 |
| Luxembourg | 65 | 65 | 65 | 65 | 65 | 65 | 65 | 65 |
| Slovenia | 65 | 65 | 65 | 65 | 65 | 65 | 65 | 65 |
| Spain | 65 | 65 | 65 | 65 | 65 | 65 | 65 | 65 |
| Sweden | 65 | 65 | 65 | 65 | 65 | 65 | 65 | 65 |
| Switzerland | 65 | 64 | 65 | 64 | 65 | 64 | 65 | 64 |

Note: Retirement age of Czech women is based on the number of children; Sweden has flexible retirement age from 61/62, but full pension age is 65.

Source: MISSOC Database. Israeli National Insurance (Israel’s National Insurance Institute, 2022).

Table A2: Additional summary statistics

| Variable | Whole sample | | | | Gender | | | | | Education | | | | |  |
| --- | --- | --- | --- | --- | --- | --- | --- | --- | --- | --- | --- | --- | --- | --- | --- |
|  |  | | | | Men | | Women | | | Low | | | High | | |
|  | Mean | SD | Min | Max | Mean | SD | | Mean | SD | Mean | SD | Mean | | SD |  |
| Wave 5 |  |  |  |  |  |  | |  |  |  |  |  | |  |  |
| Age | 64.01 | 7.33 | 48 | 78 | 64.38 | 7.18 | | 63.75 | 7.44 | 65.39 | 7.29 | 62.92 | | 7.18 |  |
| Female | 0.58 | 0.49 | 0 | 1 | 0.00 | 0.00 | | 1.00 | 0.00 | 0.60 | 0.49 | 0.56 | | 0.50 |  |
| Loneliness | 3.66 | 1.17 | 3 | 9 | 3.55 | 1.06 | | 3.75 | 1.25 | 3.72 | 1.24 | 3.62 | | 1.11 |  |
| Feeling isolated | 0.12 | 0.32 | 0 | 1 | 0.10 | 0.30 | | 0.13 | 0.34 | 0.14 | 0.34 | 0.11 | | 0.31 |  |
| Feeling left out | 0.17 | 0.37 | 0 | 1 | 0.14 | 0.35 | | 0.19 | 0.39 | 0.17 | 0.38 | 0.17 | | 0.37 |  |
| Lack companionship | 0.27 | 0.44 | 0 | 1 | 0.23 | 0.42 | | 0.30 | 0.46 | 0.29 | 0.45 | 0.25 | | 0.43 |  |
| Number of activities last year | 2.53 | 1.53 | 0 | 8 | 2.45 | 1.53 | | 2.58 | 1.53 | 2.12 | 1.48 | 2.85 | | 1.49 |  |
| Participate in group activity | 0.67 | 0.47 | 0 | 1 | 0.69 | 0.46 | | 0.65 | 0.48 | 0.58 | 0.49 | 0.73 | | 0.44 |  |
| Observations | 19,699 | 19,699 | 19,699 | 19,699 | 8,344 | 8,344 | | 11,355 | 11,355 | 8,722 | 8,722 | 10,977 | | 10,977 |  |
| Wave 6 |  |  |  |  |  |  | |  |  |  |  |  | |  |  |
| Age | 66.01 | 7.33 | 50 | 80 | 66.38 | 7.18 | | 65.75 | 7.44 | 67.39 | 7.29 | 64.92 | | 7.18 |  |
| Female | 0.58 | 0.49 | 0 | 1 | 0.00 | 0.00 | | 1.00 | 0.00 | 0.60 | 0.49 | 0.56 | | 0.50 |  |
| Loneliness | 3.73 | 1.20 | 3 | 9 | 3.61 | 1.10 | | 3.82 | 1.26 | 3.79 | 1.27 | 3.69 | | 1.14 |  |
| Feeling isolated | 0.14 | 0.34 | 0 | 1 | 0.11 | 0.32 | | 0.15 | 0.36 | 0.15 | 0.35 | 0.13 | | 0.33 |  |
| Feeling left out | 0.19 | 0.39 | 0 | 1 | 0.16 | 0.37 | | 0.21 | 0.41 | 0.19 | 0.39 | 0.19 | | 0.39 |  |
| Lack companionship | 0.30 | 0.46 | 0 | 1 | 0.25 | 0.43 | | 0.33 | 0.47 | 0.32 | 0.47 | 0.28 | | 0.45 |  |
| Number of activities last year | 2.53 | 1.51 | 0 | 7 | 2.46 | 1.53 | | 2.59 | 1.50 | 2.13 | 1.47 | 2.85 | | 1.47 |  |
| Participate in group activity | 0.66 | 0.48 | 0 | 1 | 0.67 | 0.47 | | 0.65 | 0.48 | 0.56 | 0.50 | 0.73 | | 0.44 |  |
| Observations | 19,699 | 19,699 | 19,699 | 19,699 | 8,344 | 8,344 | | 11,355 | 11,355 | 8,722 | 8,722 | 10,977 | | 10,977 |  |
| Wave 8 |  |  |  |  |  |  | |  |  |  |  |  | |  |  |
| Age | 70.49 | 7.31 | 54 | 85 | 70.85 | 7.15 | | 70.22 | 7.42 | 71.85 | 7.27 | 69.40 | | 7.16 |  |
| Female | 0.58 | 0.49 | 0 | 1 | 0.00 | 0.00 | | 1.00 | 0.00 | 0.60 | 0.49 | 0.56 | | 0.50 |  |
| Loneliness | 3.78 | 1.26 | 3 | 9 | 3.66 | 1.14 | | 3.87 | 1.33 | 3.86 | 1.36 | 3.72 | | 1.17 |  |
| Feeling isolated | 0.15 | 0.36 | 0 | 1 | 0.13 | 0.33 | | 0.16 | 0.37 | 0.17 | 0.37 | 0.13 | | 0.34 |  |
| Feeling left out | 0.20 | 0.40 | 0 | 1 | 0.18 | 0.38 | | 0.21 | 0.41 | 0.21 | 0.40 | 0.19 | | 0.39 |  |
| Lack companionship | 0.30 | 0.46 | 0 | 1 | 0.26 | 0.44 | | 0.33 | 0.47 | 0.33 | 0.47 | 0.28 | | 0.45 |  |
| Number of activities last year | 2.49 | 1.48 | 0 | 7 | 2.39 | 1.47 | | 2.57 | 1.49 | 2.10 | 1.45 | 2.81 | | 1.43 |  |
| Participate in group activity | 0.65 | 0.48 | 0 | 1 | 0.66 | 0.48 | | 0.64 | 0.48 | 0.56 | 0.50 | 0.72 | | 0.45 |  |
| Observations | 19,699 | 19,699 | 19,699 | 19,699 | 8,344 | 8,344 | | 11,355 | 11,355 | 8,722 | 8,722 | 10,977 | | 10,977 |  |

Notes: Loneliness refers to the short version of the R-UCLA loneliness scale. Feeling isolated, feeling left out and lack companionship are indicator variables equal to one if respondents felt either of these feelings “some of the time” or “often”. The number of activities refers to a specific set of activities respondents have engaged in during the last 12 months. “Participated in group activities” refers to a dummy equal to one if the respondent has engaged in at least one kind of activity classified as a group activity during the last 12 months.

Table A3: Robustness checks: full sample

|  | (1) | (2) | (3) | (4) | (5) | (6) | (7) | (8) | | (9) | | (10) | | (11) | | | (12) | |
| --- | --- | --- | --- | --- | --- | --- | --- | --- | --- | --- | --- | --- | --- | --- | --- | --- | --- | --- |
|  | Definition 2 | Definition 3 | DV: Lone  > 3 | Age dummy | age 3 | Age*country | Ages  52-72 | Ages 54-70 | | Ages 56-68 | | No never retired | | No always retired | | | No later retired | |
| Loneliness |  |  |  |  |  |  |  |  | |  | |  | |  | | |  | |
| Retired $R_{t}$ | 0.172 | 0.111 | 0.019 | 0.264 | 0.196 | 0.120 | 0.169 | 0.152 | | 0.185 | | 0.145 | | 0.161 | | | 0.115 | |
| *(short-term)* | (0.161) | (0.151) | (0.058) | (0.189) | (0.148) | (0.12) | (0.136) | (0.145) | | (0.168) | | (0.130) | | (0.128) | | | (0.110) | |
| Retired $R_{t-1}$ | -0.382*** | -0.311** | -0.112* | -0.361** | -0.296*** | -0.292*** | -0.288*** | -0.265** | | -0.315** | | -0.306*** | | -0.270*** | | | -0.249*** | |
| *(long-term)* | (0.123) | (0.125) | (0.057) | (0.153) | (0.113) | (0.097) | (0.103) | (0.111) | | (0.132) | | (0.106) | | (0.101) | | | (0.087) | |
| Isolated |  |  |  |  |  |  |  |  | |  | |  | |  | | |  | |
| Retired $R_{t}$ | 0.049 | 0.031 |  | 0.0493 | 0.0556 | 0.0432 | 0.046 | 0.032 | | 0.031 | | 0.032 | | 0.054 | | | 0.034 | |
| *(short-term)* | (0.053) | (0.052) |  | (0.062) | (0.049) | (0.0361) | (0.045) | (0.048) | | (0.056) | | (0.042) | | (0.039) | | | (0.039) | |
| Retired $R_{t-1}$ | -0.144*** | -0.115*** |  | -0.113** | -0.101*** | -0.114*** | -0.103*** | | -0.101*** | | -0.099** | | -0.103*** | | -0.0942*** | | | -0.105*** |
| *(long-term)* | (0.039) | (0.041) |  | (0.046) | (0.036) | (0.0307) | (0.033) | (0.035) | | (0.040) | | (0.033) | | (0.031) | | (0.029) | | |
| Left out |  |  |  |  |  |  |  |  | |  | |  | |  | |  | | |
| Retired $R_{t}$ | 0.015 | 0.007 |  | 0.0282 | 0.0247 | 0.002 | 0.020 | 0.011 | | 0.017 | | 0.004 | | 0.019 | | -0.002 | | |
| *(short-term)* | (0.056) | (0.055) |  | (0.070) | (0.052) | (0.041) | (0.047) | (0.051) | | (0.058) | | (0.046) | | (0.047) | | (0.037) | | |
| Retired $R_{t-1}$ | -0.066 | -0.036 |  | -0.0549 | -0.0502 | -0.0520 | -0.049 | -0.025 | | -0.051 | | -0.045 | | -0.075** | | -0.030 | | |
| *(long-term)* | (0.050) | (0.050) |  | (0.063) | (0.046) | (0.039) | (0.042) | (0.046) | | (0.053) | | (0.043) | | (0.036) | | (0.038) | | |
| Lack companionship | |  |  |  |  |  |  |  | |  | |  | |  | |  | | |
| Retired $R_{t}$ | 0.039 | 0.010 |  | 0.104 | 0.0453 | 0.027 | 0.039 | 0.045 | | 0.047 | | 0.042 | | 0.023 | | 0.034 | | |
| *(short-term)* | (0.069) | (0.069) |  | (0.077) | (0.063) | (0.053) | (0.057) | (0.061) | | (0.070) | | (0.058) | | (0.051) | | (0.048) | | |
| Retired $R_{t-1}$ | -0.109* | -0.081 |  | -0.115 | -0.0902 | -0.082 | -0.081 | -0.085 | | -0.086 | | -0.096 | | -0.059 | | -0.071 | | |
| *(long-term)* | (0.061) | (0.072) |  | (0.072) | (0.064) | (0.054) | (0.058) | (0.062) | | (0.073) | | (0.059) | | (0.049) | | (0.050) | | |
| First stage | 0.213*** | 0.224*** | 0.271^***^ | 0.224*** | 0.243*** | 0.266*** | 0.259*** | 0.248*** | | 0.227*** | | 0.263*** | | 0.401*** | | 0.303*** | | |
|  | (0.018) | (0.020) | (0.021) | (0.021) | (0.021) | (0.019) | (0.021) | (0.021) | | (0.022) | | (0.022) | | (0.029) | | (0.025) | | |
| First stage F | 135.65 | 118.32 | 173.63 | 109.77 | 135.68 | 180.83 | 150.09 | 135.27 | | 105.13 | | 142.976 | | 178.76 | | 147.314 | | |
| # Observations | 39,398 | 34,148 | 39,398 | 39,398 | 39,398 | 39,398 | 30,344 | 26,378 | | 21,044 | | 29,488 | | 18,820 | | 33,556 | | |
| # Individuals | 19,699 | 17,074 | 19,699 | 19,699 | 19,699 | 19,699 | 15,172 | 13,189 | | 10,522 | | 14,744 | | 9,410 | | 16,778 | | |

Note: Fixed effect IV estimates of the effect of retirement on (the dimensions of) loneliness in the short- and long—term (separate regressions), as well as the respective first stages for each set of regressions. Column (1) shows estimates using retirement definition 2, column (2) uses retirement definition 3, column (3) uses the main specification, but as outcome a dummy for loneliness levels higher than 3. Column (4) reports the results with age specified as age dummies. Column (5) adds a cubic age term as an additional control. Column (6) reports the results country specific age trends. Column (7) uses the main retirement definition but restricts the sub-sample to individuals aged 52-72 in wave 6, (8) uses individuals aged 54-70, and (9) individuals aged 56-68. Column (10) displays results excluding individuals who never retire in the three waves, in column (11) we exclude individuals who are always retired in the three waves, and in column (12), we exclude individuals who retire between waves 6 and 8. All regressions include control variables from equations (1) and (2). Standard errors clustered at the individual level and at the policy level (age*gender*country) are in parentheses. *** p<0.01, ** p<0.05, * p<0.1.

Table A4: Robustness checks: low education

|  | (1) | (2) | (3) | (4) | (5) | (6) | | (7) | | | | (8) | | | | (9) | | | | (10) | | | | (11) | | | | (12) | | | |
| --- | --- | --- | --- | --- | --- | --- | --- | --- | --- | --- | --- | --- | --- | --- | --- | --- | --- | --- | --- | --- | --- | --- | --- | --- | --- | --- | --- | --- | --- | --- | --- |
|  | Definition 2 | Definition 3 | DV: Lone  > 3 | Age dummy | Age 3 | Age*country | | Ages  52-72 | | | | Ages 54-70 | | | | Ages 56-68 | | | | No never retired | | | | No always retired | | | | No later retired | | | |
| Loneliness |  |  |  |  |  |  | |  | | | |  | | | |  | | | |  | | | |  | | | |  | | | |
| Retired $R_{t}$ | 0.0208 | 0.0792 | 0.0208 | 0.239 | 0.0835 | 0.00568 | | 0.0449 | | | | 0.0403 | | | | 0.0653 | | | | 0.0185 | | | | 0.0122 | | | | 0.0208 | | | |
| *(short-term)* | (0.261) | (0.295) | (0.0924) | (0.274) | (0.218) | (0.190) | | (0.203) | | | | (0.216) | | | | (0.240) | | | | (0.216) | | | | (0.160) | | | | (0.178) | | | |
| Retired $R_{t-1}$ | -0.260 | -0.255 | -0.0560 | -0.211 | -0.279 | -0.202 | | -0.222 | | | | -0.233 | | | | -0.232 | | | | -0.294 | | | | -0.237* | | | | -0.0703 | | | |
| *(long-term)* | (0.218) | (0.255) | (0.0846) | (0.259) | (0.185) | (0.176) | | (0.170) | | | | (0.182) | | | | (0.208) | | | | (0.191) | | | | (0.139) | | | | (0.146) | | | |
| Isolated |  |  |  |  |  |  | |  | | | |  | | | |  | | | |  | | | |  | | | |  | | | |
| Retired $R_{t}$ | -0.0234 | -0.0255 |  | -0.0402 | -0.00733 | | -0.00781 | | | -0.0103 | | | | -0.0243 | | | | -0.0734 | | | | -0.00461 | | | | 0.0112 | | | | -0.0180 | |
| *(short-term)* | (0.0838) | (0.0939) |  | (0.0894) | (0.0701) | | (0.0578) | | | (0.0654) | | | | (0.0714) | | | | (0.0825) | | | | (0.0705) | | | | (0.0522) | | | | (0.0576) | |
| Retired $R_{t-1}$ | -0.114 | -0.0830 |  | -0.105 | -0.0838 | | -0.0860 | | | -0.0786 | | | | -0.0861 | | | | -0.0578 | | | | -0.0917 | | | | -0.0884** | | | | -0.0634 | |
| *(long-term)* | (0.0758) | (0.0903) |  | (0.0889) | (0.0635) | | (0.0582) | | | (0.0580) | | | | (0.0618) | | | | (0.0716) | | | | (0.0653) | | | | (0.0414) | | | | (0.0554) | |
| Left out |  |  |  |  |  |  | |  | | | |  | | | |  | | | |  | | | |  | | | |  | | | |
| Retired $R_{t}$ | -0.0870 | -0.0953 |  | 0.00785 | -0.0377 | | -0.0751 | | -0.0581 | | | | -0.0572 | | | | -0.0320 | | | | -0.0747 | | | | -0.0251 | | | | -0.0730 | | |
| *(short-term)* | (0.0863) | (0.102) |  | (0.0954) | (0.0720) | (0.0648) | | (0.0678) | | | | (0.0729) | | | | (0.0781) | | | | (0.0723) | | | | (0.0508) | | | | (0.0595) | | | |
| Retired $R_{t-1}$ | -0.0129 | 0.00509 |  | -0.00318 | -0.0236 | -0.00695 | | -0.0178 | | | | -0.00881 | | | | -0.0277 | | | | -0.0251 | | | | -0.0478 | | | | 0.0230 | | | |
| *(long-term)* | (0.0808) | (0.0929) |  | (0.0940) | (0.0669) | (0.0642) | | (0.0616) | | | | (0.0668) | | | | (0.0757) | | | | (0.0701) | | | | (0.0518) | | | | (0.0542) | | | |
| Lack of companionship | |  |  |  |  |  | |  | | | |  | | | |  | | | |  | | | |  | | | |  | | | |
| Retired $R_{t}$ | 0.0595 | 0.0712 |  | 0.182 | 0.0686 | 0.0471 | | 0.0576 | | | | 0.0600 | | | | 0.0537 | | | | 0.0464 | | | | 0.0208 | | | | 0.0493 | | | |
| *(short-term)* | (0.117) | (0.131) |  | (0.118) | (0.0958) | (0.0853) | | (0.0888) | | | | (0.0938) | | | | (0.105) | | | | (0.0979) | | | | (0.0641) | | | | (0.0813) | | | |
| Retired $R_{t-1}$ | -0.159 | -0.156 |  | -0.160 | -0.167* | -0.135 | | -0.134 | | | | -0.135 | | | | -0.132 | | | | -0.158* | | | | -0.0869 | | | | -0.0785 | | | |
| *(long-term)* | (0.111) | (0.128) |  | (0.112) | (0.0926) | (0.0826) | | (0.0860) | | | | (0.0910) | | | | (0.103) | | | | (0.0912) | | | | (0.0618) | | | | (0.0774) | | | |
| First stage | 0.192*** | 0.199*** | 0.263*** | 0.217*** | 0.243*** | | 0.262*** | | | | 0.256*** | | | | 0.244*** | | | | 0.227*** | | | | 0.255*** | | | | 0.445*** | | | | 0.286*** |
|  | (0.0247) | (0.0195) | (0.029) | (0.0208) | (0.0292) | | (0.0276) | | | | (0.0292) | | | | (0.0295) | | | | (0.0302) | | | | (0.0319) | | | | (0.0425) | | | | (0.0328) |
| First stage F | 60.61 | 48.22 | 82.19 | 56.94 | 68.64 | | 88.29 | | | | 76.32 | | | | 67.88 | | | | 55.16 | | | | 64.12 | | | | 82.19 | | | | 76.27 |
| # Observations | 17,444 | 14,278 | 17,444 | 17,444 | 17,444 | 17,444 | | 12,334 | | | | 10577 | | | | 8,414 | | | | 13,648 | | | | 7,320 | | | | 15,214 | | | |
| # Individuals | 8,722 | 7,139 | 8,722 | 8,722 | 8,722 | 8,722 | | 6,167 | | | | 5,285 | | | | 4,207 | | | | 6,824 | | | | 3,660 | | | | 7,607 | | | |

Note: Fixed effect IV estimates of the effect of retirement on (the dimensions of) loneliness in the short- and long—term (separate regressions) for women, as well as the respective first stages for each set of regressions. Column (1) shows estimates using retirement definition 2, column (2) uses retirement definition 3, column (3) uses the main specification, but as outcome a dummy for loneliness levels higher than 3. Column (4) reports the results with age specified as age dummies, and (5) as age 3. Column (6) reports the results country specific age trends. Column (7) uses the main retirement definition but restricts the sub-sample to individuals aged 52-72 in wave 6, (8) uses individuals aged 54-70, and (9) individuals aged 56-68. Column (10) displays results excluding individuals who never retire in the three waves, in column (11) we exclude individuals who are always retired in the three waves, and in column (12), we exclude individuals who retire between waves 6 and 8. All regressions include control variables from equations (1) and (2). Standard errors clustered at the individual level and at the policy level (age*gender*country) are in parentheses. *** p<0.01, ** p<0.05, * p<0.1.

Table A5: Robustness checks: high education

|  | (1) | (2) | (3) | (4) | (5) | (6) | | (7) | | (8) | | (9) | | (10) | | | | (11) | | | | (12) | | | | | |
| --- | --- | --- | --- | --- | --- | --- | --- | --- | --- | --- | --- | --- | --- | --- | --- | --- | --- | --- | --- | --- | --- | --- | --- | --- | --- | --- | --- |
|  | Definition 2 | Definition 3 | DV: Lone  > 3 | Age dummy | Age 3 | Age*country | | Ages  52-72 | | Ages 54-70 | | Ages 56-68 | | No never retired | | | | No always retired | | | | No later retired | | | | | |
| Loneliness |  |  |  |  |  |  | |  | |  | |  | |  | | | |  | | | |  | | | | | |
| Retired $R_{t}$ | 0.254 | 0.123 | 0.0166 | 0.267 | 0.267 | 0.201 | | 0.248 | | 0.220 | | 0.265 | | 0.228 | | | | 0.278 | | | | 0.169 | | | | | |
| *(short-term)* | (0.183) | (0.155) | (0.0670) | (0.234) | (0.182) | (0.137) | | (0.166) | | (0.179) | | (0.214) | | (0.145) | | | | (0.195) | | | | (0.128) | | | | | |
| Retired $R_{t-1}$ | -0.414*** | -0.315** | -0.139** | -0.429** | -0.294** | -0.324*** | | -0.307** | | -0.272* | | -0.358** | | -0.299** | | | | -0.224 | | | | -0.335*** | | | | | |
| *(long-term)* | (0.154) | (0.141) | (0.0635) | (0.185) | (0.149) | (0.120) | | (0.135) | | (0.147) | | (0.172) | | (0.132) | | | | (0.146) | | | | (0.115) | | | | | |
| Isolated |  |  |  |  |  |  | |  | |  | |  | |  | | | |  | | | |  | | | | | |
| Retired $R_{t}$ | 0.0925 | 0.0598 |  | 0.105 | 0.0996 | | 0.0751 | 0.0840 | | 0.0709 | | 0.109 | | | 0.0584 | | | | 0.0889 | | | 0.0671 | | |  |  |  |
| *(short-term)* | (0.0635) | (0.0595) |  | (0.0787) | (0.0620) | | (0.0474) | (0.0572) | | (0.0610) | | (0.0718) | | | (0.0502) | | | | (0.0547) | | | (0.0489) | | |  |  |  |
| Retired $R_{t-1}$ | -0.152*** | -0.126*** |  | -0.112** | -0.109*** | | -0.122*** | -0.114*** | | -0.108*** | | -0.125*** | | | -0.106*** | | | | -0.0834* | | | -0.126*** | | |  |  |  |
| *(long-term)* | (0.0434) | (0.0422) |  | (0.0540) | (0.0411) | | (0.0357) | (0.0375) | | (0.0408) | | (0.0472) | | | (0.0370) | | | | (0.0456) | | | (0.0337) | | |  |  |  |
| Left out |  |  |  |  |  |  | |  | |  | |  | |  | | | |  | | | |  | | | | | |
| Retired $R_{t}$ | 0.0707 | 0.0545 |  | 0.0396 | 0.0659 | | 0.0480 | 0.0714 | | 0.0553 | | 0.0513 | | | | 0.0551 | | | | 0.0479 | | | 0.0402 | | | |  |
| *(short-term)* | (0.0678) | (0.0617) |  | (0.0894) | (0.0670) | (0.0506) | | (0.0611) | | (0.0658) | | (0.0783) | | (0.0561) | | | | (0.0766) | | | | (0.0457) | | | | | |
| Retired $R_{t-1}$ | -0.0822 | -0.0442 |  | -0.0825 | -0.0668 | -0.0681 | | -0.0636 | | -0.0319 | | -0.0641 | | -0.0536 | | | | -0.0721 | | | | -0.0539 | | | | | |
| *(long-term)* | (0.0588) | (0.0587) |  | (0.0821) | (0.0581) | (0.0481) | | (0.0534) | | (0.0581) | | (0.0669) | | (0.0521) | | | | (0.0547) | | | | (0.0475) | | | | | |
| Lack of companionship | |  |  |  |  |  | |  | |  | |  | |  | | | |  | | | |  | | | | | |
| Retired $R_{t}$ | 0.0260 | -0.0207 |  | 0.0442 | 0.0235 | 0.0187 | | 0.0243 | | 0.0313 | | 0.0397 | | 0.0394 | | | | 0.0246 | | | | 0.0218 | | | | | |
| *(short-term)* | (0.0853) | (0.0804) |  | (0.102) | (0.0825) | (0.0687) | | (0.0760) | | (0.0811) | | (0.0946) | | (0.0752) | | | | (0.0773) | | | | (0.0615) | | | | | |
| Retired $R_{t-1}$ | -0.0682 | -0.0368 |  | -0.0749 | -0.0296 | -0.0420 | | -0.0364 | | -0.0481 | | -0.0507 | | -0.0508 | | | | -0.0259 | | | | -0.0546 | | | | | |
| *(long-term)* | (0.0843) | (0.0781) |  | (0.0911) | (0.0804) | (0.0682) | | (0.0734) | | (0.0785) | | (0.0925) | | (0.0737) | | | | (0.0716) | | | | (0.0611) | | | | | |
| First stage | 0.226*** | 0.237*** | 0.275*** | 0.228*** | 0.243*** | | 0.273*** | | 0.260*** | | 0.250*** | | 0.226*** | | | | 0.269*** | | | | 0.351*** | | | 0.217*** | |  |  |
|  | (0.0198) | (0.0160) | (0.021) | (0.0178) | (0.0212) | | (0.0205) | | (0.0212) | | (0.0217) | | (0.0231) | | | | (0.0219) | | | | (0.0329) | | | (0.0208) | |  |  |
| First stage F | 128.11 | 116.98 | 173.68 | 89.94 | 129.88 | | 175.87 | | 146.91 | | 128.79 | | 94.12 | | | | 150.28 | | | | 173.68 | | | 143.55 | |  |  |
| # Observations | 21,954 | 19,870 | 21,954 | 21,954 | 21,954 | 21,954 | | 18,010 | | 15,808 | | 12,630 | | 15,840 | | | | 11,500 | | | | 17,444 | | | | | |
| # Individuals | 10,977 | 9,935 | 10,977 | 10,977 | 10,977 | 10,977 | | 9,005 | | 7,904 | | 6,315 | | 7,920 | | | | 5,750 | | | | 8,722 | | | | | |

Note: Fixed effect IV estimates of the effect of retirement on (the dimensions of) loneliness in the short- and long—term (separate regressions) for women, as well as the respective first stages for each set of regressions. Column (1) shows estimates using retirement definition 2, column (2) uses retirement definition 3, and column (3) uses the main specification but as outcome a dummy for loneliness levels higher than 3. Column (4) reports the results with age specified as age dummies and (5) as age 3. Column (6) reports the results country specific age trends. Column (7) uses the main retirement definition but restricts the sub-sample to individuals aged 52-72 in wave 6, (8) uses individuals aged 54-70, and (9) individuals aged 56-68. Column (10) displays results excluding individuals who never retire in the three waves, in column (11) we exclude individuals who are always retired in the three waves, and in column (12), we exclude individuals who retire between waves 6 and 8. All regressions include control variables from equations (1) and (2). Standard errors clustered at the individual level and at the policy level (age*gender*country) are in parentheses. *** p<0.01, ** p<0.05, * p<0.1.

Table A6: Robustness checks: women

|  | (1) | (2) | (3) | (4) | (5) | (6) | | (7) | | | | (8) | | | | (9) | | | | (10) | | | | (11) | | | | (12) | | | |  |
| --- | --- | --- | --- | --- | --- | --- | --- | --- | --- | --- | --- | --- | --- | --- | --- | --- | --- | --- | --- | --- | --- | --- | --- | --- | --- | --- | --- | --- | --- | --- | --- | --- |
|  | Definition 2 | Definition 3 | DV: Lone  > 3 | Age dummy | Age 3 | Age*country | | Ages  52-72 | | | | Ages 54-70 | | | | Ages 56-68 | | | | No never retired | | | | No always retired | | | | No later retired | | | |  |
| Loneliness |  |  |  |  |  |  | |  | | | |  | | | |  | | | |  | | | |  | | | |  | | | |  |
| Retired $R_{t}$ | 0.452** | 0.367* | 0.142* | 0.542* | 0.420* | 0.339* | | 0.370* | | | | 0.410* | | | | 0.432* | | | | 0.249 | | | | 0.330* | | | | 0.297* | | | |  |
| *(short-term)* | (0.205) | (0.198) | (0.076) | (0.29) | (0.237) | (0.174) | | (0.219) | | | | (0.231) | | | | (0.256) | | | | (0.192) | | | | (0.190) | | | | (0.172) | | | |  |
| Retired $R_{t-1}$ | -0.477** | -0.402** | -0.177** | -0.424** | -0.348** | -0.362*** | | -0.334** | | | | -0.324** | | | | -0.377** | | | | -0.277** | | | | -0.349** | | | | -0.279** | | | |  |
| *(long-term)* | (0.171) | (0.170) | (0.078) | (0.202) | (0.149) | (0.129) | | (0.137) | | | | (0.147) | | | | (0.167) | | | | (0.130) | | | | (0.139) | | | | (0.110) | | | |  |
| Isolated |  |  |  |  |  |  | |  | | | |  | | | |  | | | |  | | | |  | | | |  | | | |  |
| Retired $R_{t}$ | 0.094 | 0.064 |  | 0.105 | 0.0917 | | 0.0854 | | | 0.076 | | | | 0.081 | | | | 0.078 | | | | 0.040 | | | | 0.089 | | | | 0.049 | | |
| *(short-term)* | (0.083) | (0.081) |  | (0.089) | (0.072) | | (0.0532) | | | (0.066) | | | | (0.070) | | | | (0.078) | | | | (0.059) | | | | (0.059) | | | | (0.054) | | |
| Retired $R_{t-1}$ | -0.136** | -0.104 |  | -0.112 | -0.0913** | | -0.111*** | | | -0.090* | | | | -0.095** | | | | -0.095* | | | | -0.072 | | | | -0.117*** | | | | -0.074* | | |
| *(long-term)* | (0.056) | (0.059) |  | (0.057) | (0.047) | | (0.041) | | | (0.044) | | | | (0.047) | | | | (0.050) | | | | (0.045) | | | | (0.041) | | | | (0.040) | | |
| Left out |  |  |  |  |  |  | |  | | | |  | | | |  | | | |  | | | |  | | | |  | | | |  |
| Retired $R_{t}$ | 0.086 | 0.063 |  | 0.0813 | 0.0865 | | 0.0615 | | 0.074 | | | | 0.077 | | | | 0.069 | | | | 0.021 | | | | 0.074 | | | | 0.053 | | | |
| *(short-term)* | (0.097) | (0.092) |  | (0.111) | (0.083) | (0.0622) | | (0.077) | | | | (0.081) | | | | (0.089) | | | | (0.070) | | | | (0.074) | | | | (0.056) | | | |  |
| Retired $R_{t-1}$ | -0.086 | -0.047 |  | -0.0296 | -0.0550 | -0.0694 | | -0.059 | | | | -0.036 | | | | -0.052 | | | | -0.009 | | | | -0.089* | | | | -0.049 | | | |  |
| *(long-term)* | (0.070) | (0.073) |  | (0.082) | (0.060) | (0.0529) | | (0.054) | | | | (0.058) | | | | (0.066) | | | | (0.049) | | | | (0.052) | | | | (0.052) | | | |  |
| Lack companionship | |  |  |  |  |  | |  | | | |  | | | |  | | | |  | | | |  | | | |  | | | |  |
| Retired $R_{t}$ | 0.189** | 0.152* |  | 0.246** | 0.174* | 0.137* | | 0.153* | | | | 0.180* | | | | 0.190* | | | | 0.148* | | | | 0.091 | | | | 0.125* | | | |  |
| *(short-term)* | (0.094) | (0.093) |  | (0.114) | (0.098) | (0.0797) | | (0.089) | | | | (0.096) | | | | (0.106) | | | | (0.087) | | | | (0.064) | | | | (0.072) | | | |  |
| Retired $R_{t-1}$ | -0.209* | -0.178* |  | -0.229** | -0.163* | -0.156* | | -0.148* | | | | -0.161* | | | | -0.183* | | | | -0.161* | | | | -0.104* | | | | -0.120* | | | |  |
| *(long-term)* | (0.113) | (0.107) |  | (0.107) | (0.094) | (0.0800) | | (0.087) | | | | (0.092) | | | | (0.104) | | | | (0.088) | | | | (0.061) | | | | (0.070) | | | |  |
| First stage | 0.204*** | 0.225*** | 0.274*** | 0.204*** | 0.250*** | | 0.268*** | | | | 0.264*** | | | | 0.254*** | | | | 0.238*** | | | | 0.280*** | | | | 0.384*** | | | | 0.319*** | |
|  | (0.028) | (0.032) | (0.031) | (0.028) | (0.032) | | 0.031 | | | | (0.031) | | | | (0.032) | | | | (0.032) | | | | (0.034) | | | | (0.043) | | | | (0.039) | |
| First stage F | 54.25 | 48.34 | 75.38 | 54.25 | 61.48 | | 76.92 | | | | 68.39 | | | | 62.23 | | | | 53.62 | | | | 67.914 | | | | 79.057 | | | | 64.997 | |
| # Observations | 22,710 | 18,634 | 22,710 | 22,710 | 22,710 | 22,710 | | 17,574 | | | | 15,238 | | | | 12,202 | | | | 16,298 | | | | 11,354 | | | | 19,424 | | | |  |
| # Individuals | 11,355 | 9,317 | 11,355 | 11,355 | 11,355 | 11,355 | | 8,787 | | | | 7,619 | | | | 6,101 | | | | 8,149 | | | | 5,677 | | | | 9,712 | | | |  |

Note: Fixed effect IV estimates of the effect of retirement on (the dimensions of) loneliness in the short- and long—term (separate regressions) for women, as well as the respective first stages for each set of regressions. Column (1) shows estimates using retirement definition 2, column (2) uses retirement definition 3, column (3) uses the main specification, but as outcome a dummy for loneliness levels higher than 3. Column (4) reports the results with age specified as age dummies, and (5) as age 3. Column (6) reports the results country specific age trends. Column (7) uses the main retirement definition but restricts the sub-sample to individuals aged 52-72 in wave 6, (8) uses individuals aged 54-70, and (9) individuals aged 56-68. Column (10) displays results excluding individuals who never retire in the three waves, in column (11) we exclude individuals who are always retired in the three waves, and in column (12), we exclude individuals who retire between waves 6 and 8. All regressions include control variables from equations (1) and (2). Standard errors clustered at the individual level and at the policy level (age*gender*country) are in parentheses. *** p<0.01, ** p<0.05, * p<0.1.

Table A7: Robustness checks: men

|  | (1) | (2) | (3) | (4) | (5) | (6) | (7) | (8) | (9) | (10) | (11) | (12) |
| --- | --- | --- | --- | --- | --- | --- | --- | --- | --- | --- | --- | --- |
|  | Definition 2 | Definition 3 | DV: Lone  > 3 | Age dummy | Age 3 | Age*country | Ages  52-72 | Ages 54-70 | Ages 56-68 | No never retired | No always retired | No later retired |
| Loneliness |  |  |  |  |  |  |  |  |  |  |  |  |
| Retired $R_{t}$ | -0.119 | -0.142 | -0.128** | -0.0982 | -0.0811 | -0.103 | -0.067 | -0.182 | -0.154 | 0.031 | -0.078 | -0.108 |
| *(short-term)* | (0.167) | (0.178) | (0.059) | (0.239) | (0.164 | (0.165) | (0.148) | (0.161) | (0.210) | (0.174) | (0.134) | (0.138) |
| Retired $R_{t-1}$ | -0.272 | -0.212 | -0.037 | -0.190 | -0.213 | -0.231 | -0.222 | -0.184 | -0.207 | -0.332* | -0.088 | -0.214 |
| *(long-term)* | (0.173) | (0.183) | (0.083) | (0.226) | (0.170) | (0.149) | (0.151) | (0.164) | (0.214) | (0.171) | (0.149) | (0.141) |
| Isolated |  |  |  |  |  |  |  |  |  |  |  |  |
| Retired $R_{t}$ | 0.005 | 0.0002 |  | -0.0164 | 0.0116 | 0.006 | 0.013 | -0.034 | -0.038 | 0.026 | 0.0005 | 0.017 |
| *(short-term)* | (0.063) | (0.067) |  | (0.083) | (0.062) | (0.053) | (0.057) | (0.064) | (0.082) | (0.060) | (0.052) | (0.056) |
| Retired $R_{t-1}$ | -0.152*** | -0.126** |  | -0.0994 | -0.107** | -0.129*** | -0.115** | -0.106** | -0.097 | -0.138*** | -0.042 | -0.146*** |
| *(long-term)* | (0.056) | (0.058) |  | (0.080) | (0.054) | (0.0486) | (0.048) | (0.053) | (0.068) | (0.052) | (0.046) | (0.046) |
| Left out |  |  |  |  |  |  |  |  |  |  |  |  |
| Retired $R_{t}$ | -0.057 | -0.046 |  | -0.0564 | -0.0545 | -0.0559 | -0.045 | -0.078 | -0.048 | -0.014 | -0.056 | -0.072 |
| *(short-term)* | (0.058) | (0.674) |  | (0.089) | (0.059) | (0.0546) | (0.053) | (0.059) | (0.072) | (0.059) | (0.055) | (0.048) |
| Retired $R_{t-1}$ | -0.047 | -0.027 |  | -0.0657 | -0.0479 | -0.0410 | -0.041 | -0.015 | -0.061 | -0.089 | -0.054 | -0.009 |
| *(long-term)* | (0.075) | (0.072) |  | (0.091) | (0.074) | (0.0657) | (0.068) | (0.075) | (0.091) | (0.075) | (0.058) | (0.060) |
| Lack companionship | |  |  |  |  |  |  |  |  |  |  |  |
| Retired $R_{t}$ | -0.118 | -0.131 |  | -0.0580 | -0.118 | -0.0954 | -0.096 | -0.126* | -0.153 | -0.082 | -0.081 | -0.073 |
| *(short-term)* | (0.078) | (0.082) |  | (0.092) | (0.076) | (0.0669) | (0.068) | (0.074) | (0.094) | (0.076) | (0.066) | (0.063) |
| Retired $R_{t-1}$ | -0.008 | 0.013 |  | 0.0432 | 0.002 | -0.009 | -0.004 | 0.006 | 0.057 | -0.021 | 0.022 | -0.021 |
| *(long-term)* | (0.079) | (0.095) |  | (0.086) | (0.078) | (0.066) | (0.068) | (0.073) | (0.094) | (0.071) | (0.067) | (0.065) |
| First stage | 0.221*** | 0.221*** | 0.266*** | 0.225*** | 0.233*** | 0.262*** | 0.253*** | 0.239*** | 0.209*** | 0.245*** | 0.387*** | 0.283*** |
|  | (0.022) | (0.024) | (0.024) | (0.026) | (0.025) | (0.022) | (0.025) | (0.026) | (0.028) | (0.026) | (0.036) | (0.028) |
| First stage F | 100.46 | 82.24 | 119.89 | 76.33 | 86.479 | 137.86 | 101.113 | 83.92 | 54.31 | 86.300 | 116.719 | 99.108 |
| # Observations | 16,688 | 15,514 | 16,688 | 16,688 | 16,688 | 16,688 | 12,770 | 11,140 | 8,842 | 13,190 | 7,466 | 14,132 |
| # Individuals | 8,344 | 7,757 | 8,344 | 8,344 | 8,344 | 8,344 | 6,385 | 5,570 | 4,421 | 6,595 | 3,733 | 7,066 |

Note: Fixed effect IV estimates of the effect of retirement on (the dimensions of) loneliness in the short- and long—term (separate regressions) for women, as well as the respective first stages for each set of regressions. Column (1) shows estimates using retirement definition 2, column (2) uses retirement definition 3, column (3) uses the main specification, but as outcome a dummy for loneliness levels higher than 3. Column (4) reports the results with age specified as age dummies, and (5) as age 3. Column (6) reports the results country specific age trends. Column (7) uses the main retirement definition but restricts the sub-sample to individuals aged 52-72 in wave 6, (8) uses individuals aged 54-70, and (9) individuals aged 56-68. Column (10) displays results excluding individuals who never retire in the three waves, in column (11) we exclude individuals who are always retired in the three waves, and in column (12), we exclude individuals who retire between waves 6 and 8. All regressions include control variables from equations (1) and (2). Standard errors clustered at the individual level and at the policy level (age*gender*country) are in parentheses. *** p<0.01, ** p<0.05, * p<0.1.

Table A8: Main FE-IV results with inverse probability weights

| Panel A | (1) | | (2) | (3) | (4) | (5) |
| --- | --- | --- | --- | --- | --- | --- |
| Loneliness |  | Gender | | | Education | |
|  | All | | Men | Women | Low | High |
| Retired $R_{t}$ | 0.147 | | -0.096 | 0.366^*^ | 0.0401 | 0.219 |
| *(short-term)* | (0.126) | | (0.146) | (0.202) | (0.198) | (0.149) |
| Retired $R_{t-1}$ | -0.308^***^ | | -0.256* | -0.346^***^ | -0.207 | -0.345*** |
| *(long-term)* | (0.0986) | | (0.149) | (0.130) | (0.175) | (0.124) |

| Panel B | (1) | | (2) | (3) | (4) | (5) |
| --- | --- | --- | --- | --- | --- | --- |
| Isolated |  | Gender | | | Education | |
|  | All | | Men | Women | Low | High |
| Retired $R_{t}$ | 0.0441 | | 0.00809 | 0.0772 | -0.00882 | 0.0813 |
| *(short-term)* | (0.0414) | | (0.0545) | (0.0613) | (0.0637) | (0.0518) |
| Retired $R_{t-1}$ | -0.117*** | | -0.139*** | -0.0967** | -0.0882 | -0.129*** |
| *(long-term)* | (0.0313) | | (0.0475) | (0.0425) | (0.0575) | (0.0359) |

| Panel C | (1) | | (2) | (3) | (4) | (5) |
| --- | --- | --- | --- | --- | --- | --- |
| Left out |  | Gender | | | Education | |
|  | All | | Men | Women | Low | High |
| Retired $R_{t}$ | 0.0119 | | -0.0499 | 0.0677 | -0.0662 | 0.0625 |
| *(short-term)* | (0.0440) | | (0.0511) | (0.0714) | (0.0656) | (0.0545) |
| Retired $R_{t-1}$ | -0.0597 | | -0.0463 | -0.0730 | -0.0159 | -0.0749 |
| *(long-term)* | (0.0411) | | (0.0661) | (0.0531) | (0.0641) | (0.0499) |

| Panel D | (1) | (2) | (3) | (4) | (5) |
| --- | --- | --- | --- | --- | --- |
| Lack of companionship |  | Gender | | Education | |
|  | All | Men | Women | Low | High |
| Retired $R_{t}$ | 0.0307 | -0.0967 | 0.145* | 0.0396 | 0.0242 |
| *(short-term)* | (0.0529) | (0.0642 | (0.0822) | (0.0846) | (0.0691) |
| Retired $R_{t-1}$ | -0.0886* | -0.0177 | -0.154* | -0.117 | -0.0592 |
| *(long-term)* | (0.0538) | (0.0637) | (0.0808) | (0.0822) | (0.0670) |
| First stage | 0.271^***^ | 0.263*** | 0.275*** | 0.263*** | 0.275*** |
|  | (0.021) | (0.029) | (0.021) | (0.029) | (0.021) |
| First-stage F | 173.63 | 82.39 | 177.10 | 82.39 | 177.10 |
| # Observations | 39,398 | 22,710 | 16,688 | 17,444 | 21,954 |
| # Individuals | 19,699 | 11,355 | 8,344 | 8,722 | 10,977 |

Note: Estimates of the effect of retirement in the short- and long-term on the loneliness scale (panel A) the probability of feeling isolated (panel B), the probability of feeling left out (panel C), the probability of lack of companionship (panel D) for the entire sample (column 1), women (column 2) and men (column 3). All regressions include control variables based on equations (1) and (2). Standard errors clustered at the individual level at the policy level (age*gender*country) are in parentheses. *** p<0.01, ** p<0.05, * p<0.1.
